# Toward a Comprehensive Phylogenetic Reconstruction of the Evolutionary History of Mitogen-Activated Protein Kinases in the Plant Kingdom

**DOI:** 10.3389/fpls.2012.00271

**Published:** 2012-12-06

**Authors:** Philipp Janitza, Kristian Karsten Ullrich, Marcel Quint

**Affiliations:** ^1^Department of Molecular Signal Processing, Leibniz Institute of Plant BiochemistryHalle (Saale), Germany

**Keywords:** MAP kinase, phylogenetics, evolution, plant, gene family

## Abstract

The mitogen-activated protein kinase (MAPK) pathway is a three-tier signaling cascade that transmits cellular information from the plasma membrane to the cytoplasm where it triggers downstream responses. The MAPKs represent the last step in this cascade and are activated when both tyrosine and threonine residues in a conserved TxY motif are phosphorylated by MAPK kinases, which in turn are themselves activated by phosphorylation by MAPK kinase kinases. To understand the molecular evolution of MAPKs in the plant kingdom, we systematically conducted a Hidden-Markov-Model based screen to identify MAPKs in 13 completely sequenced plant genomes. In this analysis, we included green algae, bryophytes, lycophytes, and several mono- and eudicotyledonous species covering >800 million years of evolution. The phylogenetic relationships of the 204 identified MAPKs based on Bayesian inference facilitated the retraction of the sequence of emergence of the four major clades that are characterized by the presence of a TDY or TEY-A/TEY-B/TEY-C type kinase activation loop. We present evidence that after the split of TDY- and TEY-type MAPKs, initially the TEY-C clade emerged. This was followed by the TEY-B clade in early land plants until the TEY-A clade finally emerged in flowering plants. In addition to these well characterized clades, we identified another highly conserved clade of 45 MAPK-likes, members of which were previously described as Mak-homologous kinases. In agreement with their essential functions, molecular population genetic analysis of *MAPK genes* in *Arabidopsis thaliana* accessions reveal that purifying selection drove the evolution of the *MAPK* family, implying strong functional constraints on *MAPK* genes. Closely related MAPKs most likely subfunctionalized, a process in which differential transcriptional regulation of duplicates may be involved.

## Introduction

To facilitate responses to developmental and environmental cues, a significant share of plant genomes evolved to equip the plant with a large variety of intracellular signaling pathways. An important mode of signal transduction is protein modification such as phosphorylation. One highly conserved pathway of this category involves a tripartite mitogen-activated protein kinase (MAPK) signaling cascade that relays and amplifies stimuli that ultimately trigger intracellular responses. In a general model, a membrane bound receptor is activated usually by an extracellular stress or developmental stimulus and relays the signal to a MAP kinase kinase kinase (MAP3K), which in turn activates a MAP kinase kinase (MAP2K). The MAP2K then transduces the signal further by phosphorylating a MAPK. The MAPK phosphorylates specific substrate proteins such as other kinases, transcription factors, or enzymes (Khokhlatchev et al., 1998), which subsequently trigger the cellular response. The formation of MAPK cascades is thought to be mediated by scaffold proteins, which enable close proximity of the different players (Whitmarsh and Davis, 1998).

As with several other signaling pathways, each of the three MAP(K/2K/3K) levels are encoded by multigene families. In *Arabidopsis thaliana* (AT) for example, 60 MAP3Ks, 10 MAP2Ks, and 20 MAPKs have been identified (MAPK-Group et al., 2002). Many examples of functionally overlapping modules have been described (Rodriguez et al., 2010). For example, MAP2Ks may be able to activate more than one MAPK (Cardinale et al., 2002). Together, this indicates a vast extent of combinatorial possibilities to form specific MAPK cascades. While this enables signal integration, it may also cause loss of specificity due to cross-activation (McClean et al., 2007). In general, specificity can be maintained by the dynamics of protein–protein interaction and by spatio-temporal restraints, including cell type specificity and subcellular compartmentation (Rodriguez et al., 2010).

MAP3Ks and MAP2Ks primarily interact with each other, MAPKs, and scaffolds (Whitmarsh and Davis, 1998) or protein phosphatases (Keyse, 2000). MAPKs, however, determine pathway specificity by selectively recruiting substrate proteins. MAPKs are typically phosphorylated by MAP2Ks on threonine and tyrosine residues at a conserved TxY signature motif, the kinase activation loop (Chang and Karin, 2001). They can be divided into four major groups. Three of them carry a TEY, one of them a TDY signature motif. In addition, another group of proteins that exhibit sequence similarity to the MAPKs are the Mak-homologous kinases (MHKs). They may contain either TEY or TDY motifs. Whether they constitute functional MAPKs has been a matter of debate because they share similarities with MAPKs as well as cyclin-dependent kinases (MAPK-Group et al., 2002) and their biochemical properties are largely unknown. However, due to their conservation across lineages and similarity to the canonical MAPKs, these MAPK-like proteins will be included in this analysis.

Considering their gene family size and the combinatorial possibilities of MAPK cascade formation, it is no surprise that MAPK cascades are involved in a variety of different physiological and developmental processes. A hallmark of MAPK signaling seems to be their prominent role in the mediation of biotic and abiotic stress responses. On the biotic side this includes PAMP- and effector-triggered immunity as well as responses to water, salt, cold, osmotic stress, ozone, and reactive oxygene species on the abiotic side (reviewed in Rodriguez et al., 2010). In addition to conferring stress responses, MAPK cascades are an integral part of numerous developmental programs ranging from cytokinesis and cell differentiation to senescence and the regulation of phytohormone biosynthesis, signaling, and cross-talk (reviewed in Rodriguez et al., 2010).

To follow the evolutionary path of the substrate recruiting MAPK family within the green plant lineage, we systematically identified MAPKs and analyzed their molecular phylogeny in the unicellular green alga *Chlamydomonas reinhardtii* (CR) and 12 additional completely sequenced land plant species. With the exceptions of liverworts, hornworts, ferns, and gymnosperms (for which completely sequenced genomes are still lacking), this covers the major land plant lineages including bryophytes, lycophytes, and several mono- and eudicotyledonous angiosperms. Hence, this study complements previous analyses that addressed the occurrence of MAPKs in a restricted number of model organisms that has been presented primarily in past review articles (MAPK-Group et al., 2002; Hamel et al., 2006; Dóczi et al., 2012). Based on the major phylogenetic plant lineages with completely sequenced genomes, we provide a comprehensive overview of the phylogenetics and selective forces driving the molecular evolution of green plant MAPKs on the species and on the population level.

## Results and Discussion

An initial genomic survey based on the complete sequence of AT and expressed sequence tags (ESTs) of additional plant species revealed a considerable expansion of the three different levels of the MAPK signaling cascade relative to yeast or metazoan model organisms (MAPK-Group et al., 2002). Next, a comparative review of monocot and eudicot MAPKs additionally included the complete genomes of *Populus trichocarpa* (PT) and *Oryza sativa* (OS) and reported 21 MAPKs for poplar and 15 MAPKs for rice (Hamel et al., 2006). Therefore, the size of the MAPK protein families in the latter two species is comparable to the 20 previously identified MAPKs in AT. To draw a more detailed picture of the molecular evolution of MAPKs in green plants, we significantly extended the species range. We aimed to reconstruct the phylogeny of this gene/protein family based on 13 completely sequenced species that cover the most important land plant lineages (with the exceptions mentioned above) and the unicellular green alga CR.

### Identification of MAPKs in green plant genomes

To identify MAPKs, we performed a Hidden-Markov-Model (HMM) based search (Eddy, 2011) for similar peptide sequences in the genomes of the green alga CR, the bryophyte *Physcomitrella patens* (PP), the lycophyte *Selaginella moellendorffii* (SM), the eudicots AT, *Arabidopsis lyrata* (AL), *Carica papaya* (CP), *Vitis vinifera* (VV), PT, and *Glycine max* (GM), and the monocots OS, *Sorghum bicolor* (SB), *Brachypodium distachyon* (BD), and *Zea mays* (ZM). We identified a total of 204 canonical MAPKs with the conserved TxY signature motif in the 13 investigated genomes (Table 1). This included the vast majority of previously published MAPKs and additional ones for some species (e.g., OS, ZM), suggesting that the HMM based search effectively identified MAPKs in the assessed genomes. In addition, we identified five proteins with an atypical MEY-type activation loop. Slight discrepancies in the exact number of MAPKs identified in a subset of species that were also the subject in another recent study (Dóczi et al., 2012) are to be expected because of the different identification approaches applied. Together, the HMM based approach provided a solid basis for the subsequent phylogenetic analysis. The green alga CR (6) and the lower land plants PP (9) and SM (6) apparently evolved a smaller set of MAPKs relative to the flowering plants (12–31) indicating an expansion of this family after the divergence of flowering plants from the SM specific tracheophyte lineage.

**Table 1:** **Identification of MAP Kinases in plant genomes**.

Organism	MAPKs				MAPK-likes			published
	TEY	TDY	MEY	total	TEY	TDY	total	
*Chlamydomonas reinhartdii*	3	3	–	6	1	3	4	5^a^, 8^b^
*Physcomitrella patens*	6	2	1	9	1	4	5	10^b^
*Selaginella moellendorffii*	3	3	–	6	1	1	2	10^b^
*Zea mays*	7	13	1	21	3	1	4	17^c^
*Sorghum bicolor*	5	10	1	16	2	1	3	–
*Brachypodium distachyon*	6	9	1	16	2	1	3	–
*Oryza sativa*	5	10	1	16	4	1	5	15^a^, 17^d^
*Vitis vinifera*	7	5	–	12	–	2	2	12^e^
*Glycine max*	17	14	–	31	4	–	4	–
*Populus trichocarpa*	12	9	–	21	2	–	2	21^a^
*Carica papaya*	6	4	–	10	–	4	6	–
*Arabidopsis thaliana*	12	8	–	20	3	–	3	20^a^, 23^f^
*Arabidopsis lyrata*	12	8	–	20	2	–	2	–

*^a^Hamel et al. (2006), ^b^Rodriguez et al. (2010), ^c^Ling et al. (2010), ^d^Rohila and Yang (2007), ^e^Hyun et al. (2010), ^f^Tena et al. (2001)*.

The most pronounced expansion of the MAPK family apparently occurred in the eudicot GM, which contains 35 MAPKs. However, this may likewise be caused by the recent emergence of duplicates that could be eradicated from the GM genome. Gene losses on the other hand seem to be rather scarce, which may reflect the essential functions of most MAPKs. Taking into account that the genomes of other eukaryotic model systems generally contain less canonical TxY-type MAPKs (Chen et al., 2001; Li et al., 2011), it seems that this family gradually expanded within the plant kingdom from green algae and bryo-/lycophytes to flowering plants. Interestingly, this pattern has been observed for other substrate recruiting signaling gene families such as F-box proteins (Gagne et al., 2002; Schumann et al., 2011), that sometimes dramatically expanded in the plant lineage.

Using the MAPK profile of the HMM search, we also identified 45 MHK genes/proteins (Table 1), that had previously been described in AT. Since MHKs (from hereon referred to as MAPK-likes) showed sequence and possibly also functional similarities to the MAPKs (Garrido et al., 2004), this was to be expected. The size of this MAPK-like family (ranging from 2 to 6 members) was considerably smaller than the MAPK family, but MAPK-likes could be identified in each of the 13 investigated genomes indicating an essential and conserved function in green plants.

### Toward a phylogenetic reconstruction of the evolutionary history of MAPKs

**Figure 1:**
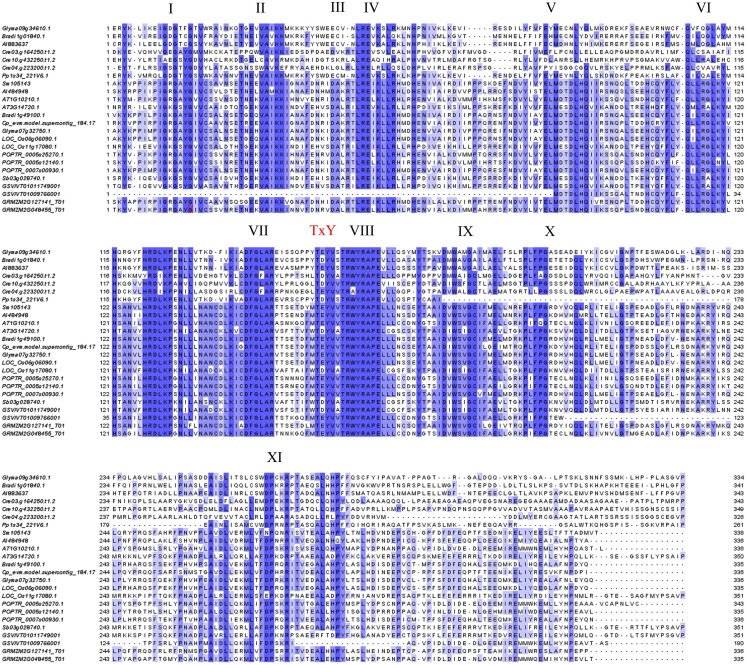
**Protein alignment of representative MAPKs from various plant species**. Twenty-four proteins representing the diversity of MAPKs in the plant kingdom were aligned using MAFFT (Katoh et al., 2005) and illustrated in Jalview (Waterhouse et al., 2009). Conserved kinase domains and the TxY activation loop are indicated. Blue color represents percent sequence conservation.

**Figure 2:**
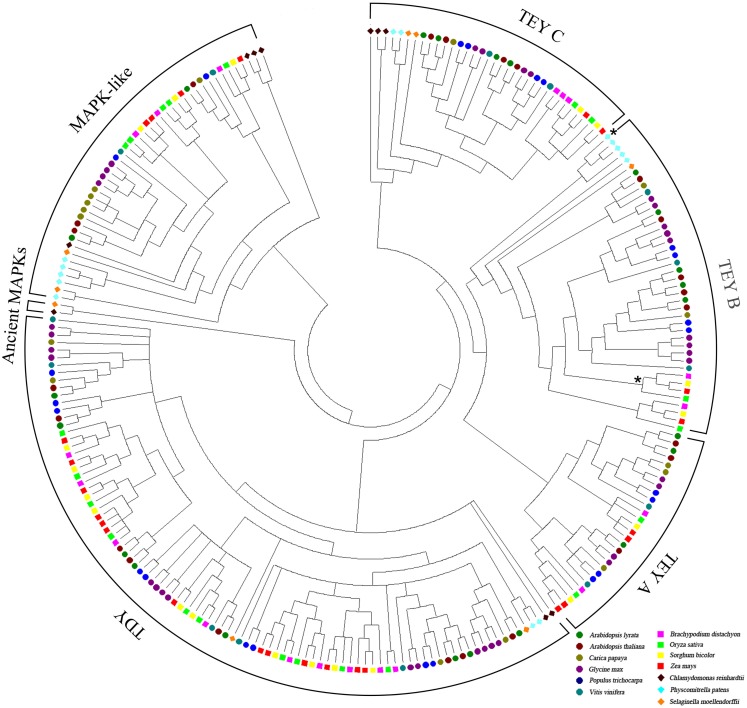
**Phylogeny of plant MAPKs and MAPK-likes**. The phylogenetic tree was created with the full-length protein sequences of 13 plant species based on Bayesian inference. The colored icons correspond to the different species. Support values/posterior probabilities and branch lengths can be found in the linear version of this tree in Figure S1 in Supplementary Material. The monocot clade and the PP sequence with the atypical MEY activation loop within the TEY-B clade are marked by asterisks.

To trace the evolutionary path of the MAPK and MAPK-like families in plants, we reconstructed the phylogenetic relations among the 249 genes/proteins we identified. The multiple sequence alignment of representative species showed a high conservation especially for the kinase activation loop, but also for most of the 11 kinase domains present in each protein (Figure 1). Based on the complete alignment, we generated a phylogenetic tree using Bayesian inference of phylogeny (Ronquist and Huelsenbeck, 2003). The resulting tree was rooted between the MAPKs and the MAPK-likes and showed a well resolved topology with posterior probabilities that greatly support the vast majority of the identified clades (Figure 2 and Figure S1 in Supplementary Material). Furthermore, it also seemed that the major clades share exon-intron structure similarities (Figure S1 in Supplementary Material). We conclude that the generated phylogeny represents a realistic picture of the evolutionary history of MAPKs and MAPK-likes.

### Evolutionary emergence of the major MAPK subfamilies

Dóczi et al. (2012) recently identified a single canonical MAPK in the basal eukaryote *Naegleria gruberi*, which – like animals and the major clade of fungi – belongs to an early diverging Heterolobosea clade. This MAPK was of the TEY-type. Provided that other basal lineages also contain only TEY-type MAPKs, this would suggest that the common ancestor of eukaryotic MAPKs may have been of this type and the distinct classes of TxY motifs have diverged from it separately in plants and animals (Dóczi et al., 2012).

Since all analyzed species in our tree contain both TEY- and TDY-type MAPKs, an early duplication event most likely generated the split between the two major canonical MAPK types. The TEYs then diverged into TEY-C and TEY-A/B, which subsequently split into TEY-A and TEY-B. This grouping into three TEY-type groups A, B, and C is already apparent on the single species level and has been described previously (MAPK-Group et al., 2002). Furthermore, the five atypical MEY-type proteins we identified all fall into the TEY-B clade. Four of these proteins are closely related monocot sequences and one is from the moss PP (marked by asterisks in Figure 2). However, the PP MEY protein is truncated and a potential MAPK function therefore rather uncertain.

Inclusion of major branches of the plant phylogeny in this tree facilitates precise tracking of the sequence from which each of the three TEY groups may have emerged. While all flowering plants evolved TEY-A, -B, and -C, the green alga CR lacks TEY-A and -B. The lower land plants PP and SM lack only TEY-A. Hence, TEY-C, which is present in all assessed plant species, most likely represents the ancient type of the TEY clade. Next, land plants evolved TEY-B, followed by the TEY-A clade finally emerging in flowering plants.

Supported by solid posterior probabilities, the MAPK-like clade is clearly separated from the canonical MAPKs and contains three basal sequences from CR that may be direct descendants of the founding MAPK-like sequence in the plant lineage (Figure S1 in Supplementary Material). Here as well, it seems that recent lineage-specific duplications cause species specific clusters such as in PP, CP, or GM.

### Nomenclature of plant MAPKs

The nomenclature of plant MAPKs has been a matter of debate since the first genome-wide survey in AT (MAPK-Group et al., 2002). Hamel et al. (2006) tried to modify the nomenclature based on the then novel plant genomes of OS and PT with the aim to enable the correct assignment of orthologous genes between flowering plant species. However, the authors still kept the original numbering from AT. What seemed to work for those three flowering plant species could not be applied to lower land plants. Dóczi et al. (2012) combined phylogenetic analyses with information from the ortholog database PLAZA (Proost et al., 2009), but failed to implement a one-to-one orthologous relation between AT and lower land plant MAPKs.

The major drawback of the present nomenclature is its focus on the MAPK set present in AT. Orthologs and paralogs in other species are currently assigned accordingly. However, our extensive phylogenetic tree revealed several Arabidopsis specific clades which clearly reflect the paralogous state of these genes (Figure 2; Figure S2 in Supplementary Material). Hence, the approximate accordance of the MAPK family sizes among plants is not indicative of synonymous gene family organization. This becomes evident when species specific duplications, for example in GM (in the TDY and TEY-A clades) or PP (in the TEY-A clade), are taken into consideration (Figure 2; Figure S2 in Supplementary Material). Yet, since the current nomenclature is based on AT, each AT paralog still has a different number lifting it to the same level as orthologous genes between other species. In conclusion, our extensive phylogenetic tree supports Dóczi et al. (2012) regarding the challenge to expand the currently used nomenclature, which is inconsistent. Hence, the only reasonable and consistent nomenclature that includes information about the ortho-/paralogy status of the genes would require rigorous renaming also of the AT MAPKs, which would require a consensus of the MAPK research community.

### Population genetic signatures of MAPKs

Classic multi-step signaling pathways or modules usually contain components that either (i) primarily relay and/or amplify the signal by interacting with other conserved signaling components, or (ii) selectively recruit target proteins that then determine the specific cellular response. The latter are frequently represented by components at the beginning of the pathway, such as ligand receptors, or those at the end of the pathway by interacting with specific response elements. In the MAPK cascade the MAPKs would be conferring downstream specificity, while MAP3Ks and MAP2Ks relay/amplify the signal.

In case the specificity determining components confer adaptive capabilities, such as disease resistance genes, they often carry signatures of positive selection and are frequently subject to birth-and-death evolution (Bakker et al., 2006). In case these signaling proteins confer functions that are essential for the plant’s life cycle and possibly conserved in a complete phylogenetic lineage, they would rather be under purifying selective pressures and interact with other conserved substrate proteins. Phylogenetic studies on F-box genes/proteins, another typical specificity determining protein family at the end of the E1-E2-E3 module in the ubiquitin-proteasome signaling pathway, showed both genes conserved across lineages and species specific genes (Hua et al., 2011; Schumann et al., 2011). Consequently, these two classes also showed contrasting signatures of selection. Purifying selection acting on conserved F-box genes and positive selection acting on recently duplicated genes specific to certain species.

For example, in contrast to F-box proteins, the number of species specific duplications of MAPKs is rather low. The vast majority of MAPKs seems to be essential from a functional perspective. We therefore aimed to analyze whether this pattern could be confirmed on the population genetic level. If MAPKs have evolved to recognize similar targets across species, one would expect purifying selection to act on the underlying genes. To examine whether these expectations were realistic, we determined sequence divergence and sequence polymorphisms for AT *MAPKs* and *MAPK-likes*. While *K*_a_/*K*_s_ ratios represent the selective pressure acting on the AT genes since their split from the sister species AL (sequence divergence), π_a_/π_s_ ratios describe selective forces acting within the AT population (sequence polymorphism).

**Figure 3:**
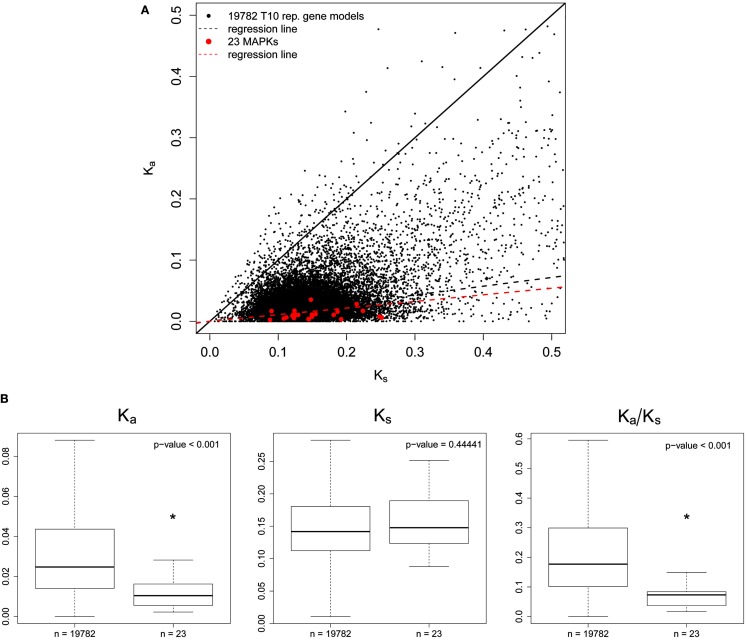
**Sequence divergence of canonical *MAPKs* and *MAPK*-likes between *Arabidopsis thaliana* and *Arabidopsis lyrata***. **(A)** *K*_a_ vs. *K*_s_ scatterplot for all 23 AT *MAPKs* and *MAPK-likes* (red dots) and 19782 TAIR10 AT representative gene models used as background data set (black dots). After identification of the closest ortholog for each of the 23 target and 19782 background genes, orthologous genes were codon-aligned and *K*_a_ and *K*_s_ values were computed. The solid black line represents neutral evolution (*K*_a_ = *K*_s_); the dashed black line represents the regression line for the background data set; the dashed red line represents the regression line for the *MAPKs/MAPK-likes*. **(B)** Box plot for *K*_a_, *K*_s_, and *K*_a_/*K*_s_ values of the 20 canonical *MAPKs* and the three *MAPK-likes* in comparison to the background data set. Medians are marked by solid black lines, boxes contain all data points in the 25–75% quartile range and whiskers denote 1.5x interquartile distances. Asterisks indicate significant differences (Wilcox test).

**Figure 4:**
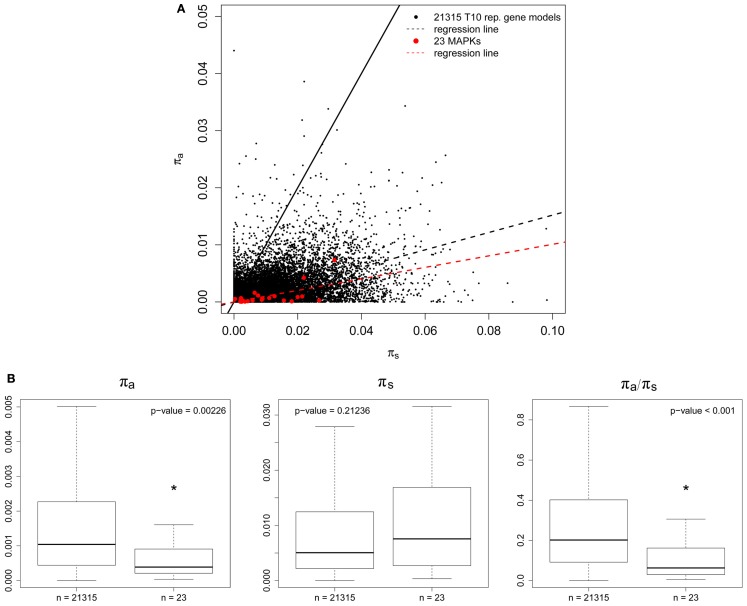
**Sequence polymorphism of canonical *MAPKs* and *MAPK*-likes among 80 natural accessions of *Arabidopsis thaliana***. **(A)** π_a_ vs. π_s_ scatter plot for all 23 AT *MAPKs* and *MAPK-likes* (red dots) and 21315 TAIR10 AT representative gene models used as background data set (black dots). The solid black line represents neutral evolution (π_a_ = π_s_); the dashed black line represents the regression line for the background data set; the dashed red line represents the regression line for the *MAPKs/MAPK-likes*. **(B)** Boxplot for π_a_, π_s_, and π_a_/π_s_ values of the 20 canonical *MAPKs* and the three *MAPK-likes* in comparison to the background data set. Medians are marked by solid black lines, boxes contain all data points in the 25–75% quartile range and whiskers denote 1.5x interquartile distances. Asterisks indicate significant differences (Wilcox test).

Calculation of *K*_a_/*K*_s_ ratios for AT *MAPKs* and *MAPK-likes* first required the identification of orthologs in the genome of the close relative AL. Figure 3A shows a *K*_a_ vs. *K*_s_ scatterplot of all orthologs we identified. The *MAPK* regression line (includes *MAPKs* and *MAPK-likes*) was below the background data set consisting of 19782 representative gene models from the TAIR10 annotation, which indicates that *MAPKs* seem to be highly conserved (Figure 3A). Comparing the *MAPK*/*MAPK-likes* family as a whole to the background data set then revealed significant differences for *K*_a_/*K*_s_ ratios and *K*_a_ values alone, but not for *K*_s_ values (Figure 3B). We find in Figure 4, that exactly the same picture emerges when we perform a similar analysis on the population level by comparing the nucleotide diversities π within 80 resequenced natural accessions of AT (Cao et al., 2011). However, *AtMPK2/AT1G59580* and *AtMPK14/At4G36450* contained premature STOP codons in two and one of the 80 assessed accessions that most likely render the remaining coding sequence (CDS) non-functional. Both genes duplicated recently and only retained orthologs in AL, indicating that these genes may be dispensable, while the sister gene retained the conserved function (clade TEY-C in Figure S1 in Supplementary Material, Figure 5A). Apart from these potential exceptions, for the CDS as a whole we observed strong signatures of purifying selection in agreement with the necessity to evolutionary conserve the majority of *MAPKs*/*MAPK-likes* because of their essential biological functions.

**Figure 5:**
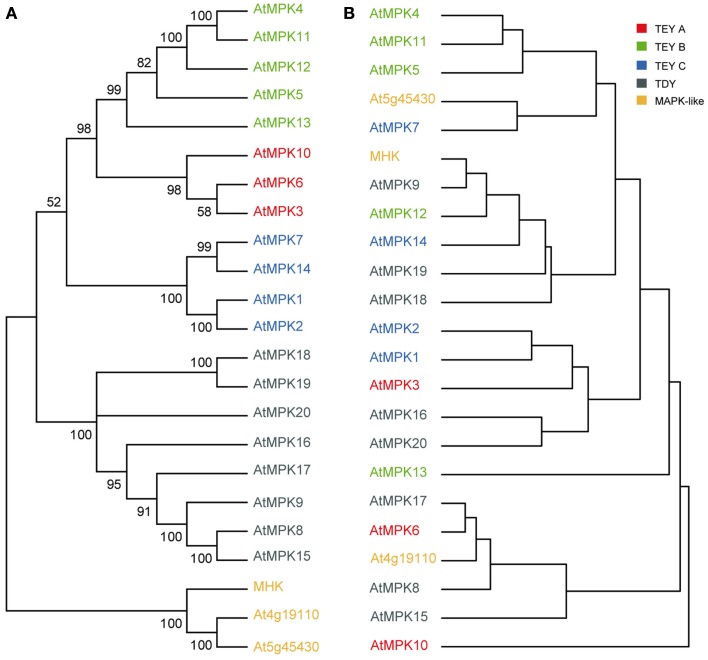
**Differential transcription profiles of phylogenetically closely related *MAPKs* in *Arabidopsis thaliana***. **(A)** Neighbor joining tree of the 20 canonical MAPKs and the three MAPK-likes. Support values as measured by 1000 bootstrap iterations are indicated. **(B)** Cladogram based on co-expression data from the AtGenExpress tissue series including all 20 canonical *MAPK*s and all three *MAPK-likes* of AT. **(A,B)** The four major clades of canonical MAPKs (TDY, TEY-A, TEY-B, TEY-C) and the MAPK-likes are indicated by different colors.

We next performed sliding window analysis for both parameters, *K*_a_/*K*_s_ and π_a_/π_s_, on the same genes to identify potential sites of positive selection that may not have been recognized when assaying the CDS as a whole. Here, we find most domains to be highly conserved with frequent isolated sites showing spikes in *K*_a_/*K*_s_ and/or π_a_/π_s_ ratios (Figure S2 in Supplementary Material). These spikes potentially represent sites under positive selective pressure. A very limited number of these sites display spikes for both parameters. Most genes contain regions with significantly increased *K*_a_/*K*_s_ ratios, indicating sequence divergence between the two species AT and AL. However, these regions of increased divergence never fall into any of the conserved kinase domains or the activation loop. Within the AT population *MAPKs* and *MAPK-likes* seem to be highly conserved across the complete coding region as suggested by π_a_/π_s_ ratios ≪1. The only exception is the *MAPK-like AT5G45430*, which is highly variable between AT accessions (Figure S2 in Supplementary Material). While in F-box proteins the F-box and protein–protein interaction domains showed contrasting patterns (Schumann et al., 2011), we did not observe anything similar across the *MAPK* family in AT.

### Expression patterns of MAPKs in *Arabidopsis thaliana*

A major mode of gene family expansion is gene duplication by various mechanisms. After duplication, the duplicated daughter genes usually either subfunctionalize or one of them pseudogenizes. Given the results presented so far, we have to assume that even phylogenetically closely related MAPKs are essential and therefore non-redundant. Since we did not detect evidence for pseudogenization within AT *MAPKs*, it seems most likely that closely related *MAPKs* subfunctionalize. To address a possible mode of subfunctionalization, we made use of the publicly available AtGenExpress data (Schmid et al., 2005; Toufighi et al., 2005) and assessed whether transcriptional regulation may play a role in *MAPK* subfunctionalization. Based on their co-expression profiles across numerous developmental stages, we grouped the 20 canonical *MAPKs* and the three *MAPK-likes* from AT into different clades. This cladogram was then compared with the phylogenetic relationships of the respective genes based on an AT specific phylogeny (Figure 5). Obviously, the topologies of the two trees have very little overlap. It seems that phylogenetically closely related *MAPKs* do not share the same relatedness on a transcriptional level, where they tend to fall into different groups. We observed a similar phenomenon when we use the biotic and abiotic stress response data sets (Toufighi et al., 2005; Kilian et al., 2007; data not shown). We therefore conclude that transcriptional regulation possibly contributes to subfunctionalization of duplicated *MAPKs*. In addition, subfunctionalization may be achieved on the post-translational level by the dynamics of protein–protein interaction and by spatio-temporal restraints, including cell type specificity and subcellular compartmentation (Rodriguez et al., 2010).

## Conclusion

To understand the evolutionary history of the MAPK module within the three-tier MAPK signaling cascade we analyzed their phylogenetic relationships among 13 genomes, which represent major lineages within the plant kingdom. Reconstruction of the MAPK phylogeny across plant lineages facilitated several conclusions regarding their molecular evolution. Possibly, some of these conclusions might need to be refined once complete genome sequence information becomes available for liverworts, hornworts, ferns, and gymnosperms. Despite the absence of complete genome information for these lineages we were able to retrace the path of emergence of the four major MAPK clades TDY, TEY-A, TEY-B, and TEY-C. We furthermore continuously identified proteins with high sequence similarity to the canonical MAPKs. These poorly described MAPK-like proteins were highly conserved and present in all assessed plant genomes. They form a distinct clade in the MAPK phylogeny, but it is unclear whether they may have been derived directly from an ancestral type of canonical MAPKs or vice versa. Generally, the canonical MAPK family has gradually increased in size in the plant lineages with a notable increase in the flowering plants. Hence, at the level of deep phylogeny we find signatures of strong functional conservation. Together with complementary analyses at the population level, these data provide evidence for purifying selection acting on the MAPKs. Taking into consideration that a hallmark of MAPK function are roles in biotic and abiotic stress responses, this may seem surprising. Both biotic and abiotic stressors are usually seen as rather variable cues that are subject to change due to the changing environment and/or the composition of and co-evolution with pathogenic interactors. However, this makes sense in that the MAPK cascade is used as an intermediary module in the signal transduction cascade triggered by biotic and abiotic stimuli that usually include specificity conferring signaling components up- and/or downstream of the MAPK cascade.

## Materials and Methods

### Identification of MAPKs in 13 different plant genomes

The annotated version of peptide sequences from each of the genomes was obtained from Phytozome V 7.0 (Goodstein et al., 2012): AL (JGI release v1.0; Hu et al., 2011), AT (TAIR release 10; Swarbreck et al., 2008), BD (JGI 8x assembly release v1.0; The International Brachypodium Initiative, 2010), CP (ASGPB release of 2007; Ming et al., 2008), CR (Augustus update 10.2; Merchant et al., 2007), GM (JGI Glyma1.0; Schmutz et al., 2010), OS (MSU Release 6.0; Ouyang et al., 2007), PP (JGI assembly release v1.1; Rensing et al., 2008), PT (JGI assembly release v2.0; Tuskan et al., 2006), SM (JGI v1.0 assembly; Banks et al., 2011), SB (Sbi1.4; Paterson et al., 2009), VV (March 2010 12X assembly; The French–Italian Public Consortium for Grapevine Genome Characterization, 2007), and ZM (release 5a.59; Schnable et al., 2009).

The 20 published MAPK protein sequences from AT (MAPK-Group et al., 2002) were aligned using the L-INS-i option in MAFFT (Katoh et al., 2005). This alignment was used to generate an HMM model using the program hmmbuild from the HMMER program suite (Eddy, 2011). The HMM model was further improved by calculating HMM parameters with the hmmcalibrate package (Eddy, 2011). Using hmmsearch, the HMM model was applied in a search against the most recent protein annotations from each plant genome. MAPK identity within the obtained sequences was determined based on the presence of the activation loop followed by a kinase domain with the typical motif TxYVxxRWYRAPE or the atypical motif MEYxxRWYRAPE that had been described for OS (Rohila and Yang, 2007). Based on the proteins identified with the AT profile, we then generated species specific MAPK profiles and repeated the procedure to identify additional MAPKs.

### Alignments of all identified MAPK sequences and phylogenetic reconstruction

All identified MAPK/MAPK-like protein sequences were aligned using the L-INS-i option in MAFFT (Katoh et al., 2005) and edited manually. The JTT + G model was selected as the best fitting amino acid substitution model according to the Bayesian Information Criterion in ProtTest (Darriba et al., 2011). To reconstruct the phylogeny we used MrBayes 3.1 (Ronquist and Huelsenbeck, 2003) as implemented at the CIPRES portal (Miller et al., 2010) and initiated two runs of eight Markov-chain Monte Carlo (MCMC) chains of 2 × 10^7^ generations each from a random starting tree, sampling every 1000 generations (additional settings: Rates = gamma, Ngammacat = 4, Aamodelpr = JTT, Temp = 0.006). A 25% burn-in was chosen based on Tracer outputs (v. 1.5)^1^ and convergence was assessed by standard deviation of split frequencies falling below 0.01 and with AWTY (Nylander et al., 2008) by comparing the estimated posterior distributions of branch support from the two independent MCMC runs. The phylogenetic tree was rooted between the MAPKs and the MAPK-likes and visualized with the MEGA Tree Explorer (Tamura et al., 2011).

### Population genetic analyses

#### Generation of background data set for nucleotide diversities

The sequence data for the 80 accessions (Cao et al., 2011) was obtained from the 1001 Genomes project web site^2^. For each of the 80 accessions the filtered variant.txt.gz file, containing the positions and annotations of single nucleotide polymorphisms (SNPs) and 1–3 bp deletions for the TAIR10 release, was downloaded from http://1001genomes.org/data/MPI/MPICao2010/releases/current/strains/ (as of 2012-05-06) and the genomic sequence of the published genome of AT (TAIR10)^3^ was repalaced by the filtered variant.txt.gz entries with a custom R script and the Biostrings R package resulting in 80 pseudochromosome sequences.

The TAIR10 Genetic Feature Format Version 3 (GFF3) file^4^ was parsed with a custom GFF3 R parser for ”protein coding genes” resulting in 27206 hits for the five *A. thaliana* chromosomes without the chloroplast and mitochondrium entries. For each of the 27206 hits the CDS positions for the representative gene model^5^ were obtained from the TAIR10 GFF file. The CDS positions were then used to extract the corresponding sequences from the 80 pseudochromosomes and concatenated to 27206 multiple sequence alignments. To account for large effect SNPs, the genes predicted to have drastic effects were excluded from further analysis (mentioned in Table 2 in Supplementary Material of Cao et al., 2011). Among these major SNPs containing genes two *MAPKs* were found (*AtMPK2/AT1G59580* and *AtMPK14*/*AT4G36450*). To complete the analysis for the *MAPK* genes, for these two genes the affected accessions (Istisu-1 and Lerik1-3; Nie1-2) were excluded from the sequence alignments and the reduced alignments could be included in the within and between species analysis. In total, the 27206 representative gene models were reduced to 21338 models including all AT *MAPKs*.

#### Calculation of nucleotide diversities

The nucleotide diversity of the 21338 CDS alignments for coding, synonymous and non-synonymous sites were calculated with polydNdS using the “-A” option (Thornton, 2003) according to Tajima (1983).

#### Calculation of nucleotide divergence rates (*K*_a_/*K*_s_)

*K*_a_/*K*_s_ ratios were calculated according to Quint et al. (2012) except for using KaKs-Calculator (Zhang et al., 2006) according to Yang and Nielsen (2000) to compute the nucleotide divergence on codon alignments.

#### Sliding window analysis

The codon alignments of the *MAPKs* between TAIR10 Col-0 sequence and best blastp hit in AL were used to generate sequence alignments containing the 80 accessions (78 for AT1G59580; 79 for AT4G36450) as the ingroup and the *A. lyrata* sequence as the outgroup sequence to perform a sliding window analysis (width: 51, jump: 9) with DnaSPv5.1 (Librado and Rozas, 2009).

#### Correlation analysis of expression data

To create the dendrogram for the cluster analysis of the expression data, the R package pvclust (Suzuki and Shimodaira, 2006) was used. The expression data of the AT *MAPKs* were extracted from the AtGenExpress extended tissue series (Schmid et al., 2005). All *MAPKs* were represented on the ATH1 microarray. In pvclust, a hierarchical clustering was performed using the Pearson correlation as a similarity measurement [dist = 1−cor(*x*, *y*)] between the expression of the genes and the UPGMA method as cluster distance function.

## Conflict of Interest Statement

The authors declare that the research was conducted in the absence of any commercial or financial relationships that could be construed as a potential conflict of interest.

## Supplementary Material

The Supplementary Material for this article can be found online at http://www.frontiersin.org/Plant_Proteomics/10.3389/fpls.2012.00271/abstract

Supplementary Table S1 **Major types of MAPKs in plant genomes**. Genes may have multiple names according to different publications.

Supplementary Figure S1 **Linear phylogeny of plant MAPKs and MAPK-likes**. Linear display of the circular Bayesian tree in Figure 2. The colored icons correspond to the different species. Posterior probabilities are indicated at the respective nodes. The monocot clade and the PP sequence with the atypical MEY activation loop within the TEY-B clade are marked by asterisks. Exon-intron structures of that part of each gene that was in the alignment were plotted next to each gene; blue boxes, exon; red boxes, 5′-UTR; yellow boxes, 3′-UTR.

Supplementary Figure S2 **Sliding window plots of sequence divergence between *MAPKs* and *MAPK-likes* in *Arabidopsis thaliana* and *Arabidopsis lyrata***. For sliding window analysis, nucleotide sequences of AT (indicated by Arabidopsis Genome Initiative identifier) and homologous nucleotide sequences of AL were used. Window size was 51 bp, and step size was 9 bp. The black arrows in the plots denotes the position of the TxY activation loop.

## Footnotes

^1^http://tree.bio.ed.ac.uk/software/tracer/

^2^http://www.1001genomes.org

^3^ftp://ftp.arabidopsis.org/home/tair/Genes/TAIR10_genome_release/TAIR10_chromosome_files/TAIR10_chr_all.fas; as of 2012-05-06

^4^ftp://ftp.arabidopsis.org/home/tair/Genes/TAIR10_genome_release/TAIR10_gff3/TAIR10_GFF3_genes.gff; as of 2012-05-06

^5^ftp://ftp.arabidopsis.org/home/tair/Genes/TAIR10_genome_release/TAIR10_gene_lists/TAIR10_representative_gene_models; as of 2012-05-06
