# Supplementary material for: Toward a Comprehensive Phylogenetic Reconstruction of the Evolutionary History of Mitogen-Activated Protein Kinases in the Plant Kingdom
Source: Front Plant Sci. 2012 Dec 6;3:271. doi: 10.3389/fpls.2012.00271 (PMC3515877; doi:10.3389/fpls.2012.00271)
Supplement: Supplementary Table S1 — Major types of MAPKs in plant genomes. Genes may have multiple names according to different publications. [file 33640_Quint_DataSheet1.PDF]

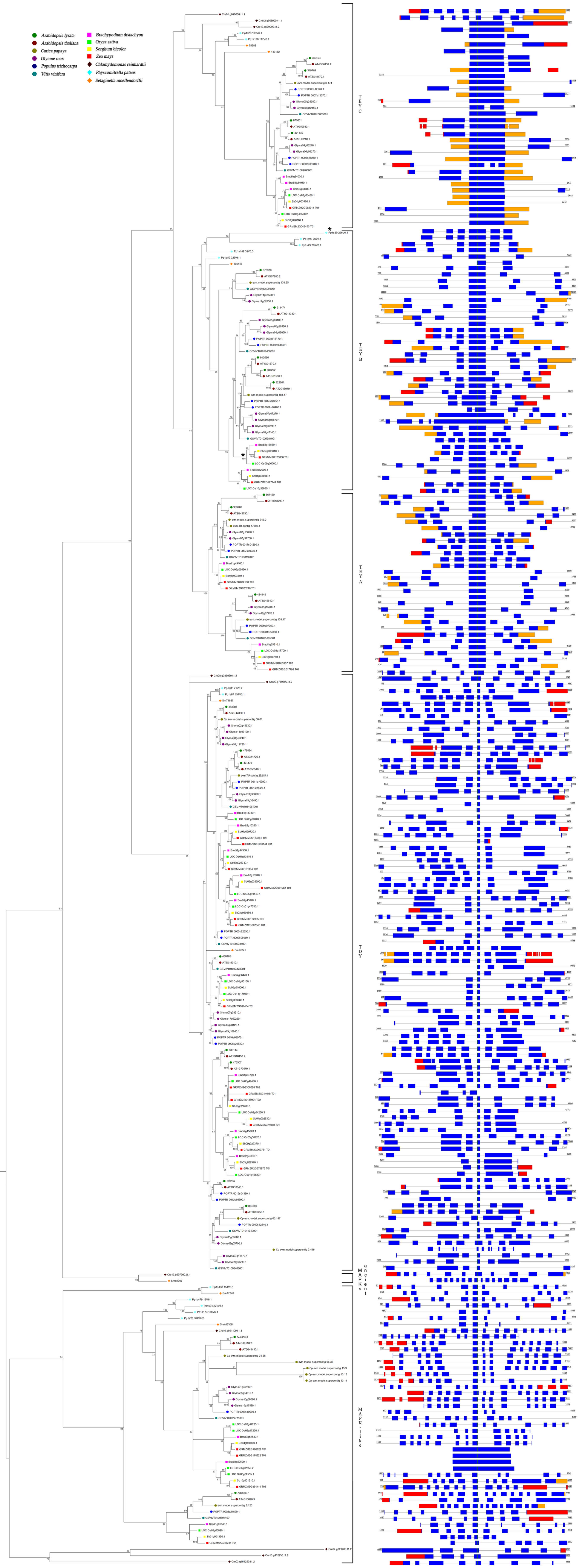

Arabidopsis lyrata
Arabidopsis thaliana
Carica papaya
Glycine max
Populus trichocarpa
Vitis vinifera
Brachypodium distachyon
Oryza sativa
Sorghum bicolor
Zea mays
Chlamydomonas reinhardtii
Physcomitrella patens
Selaginella moellendorffii
TEYC
TEYB
TEYA
TDY
MAPKs ancient
MAPK-like

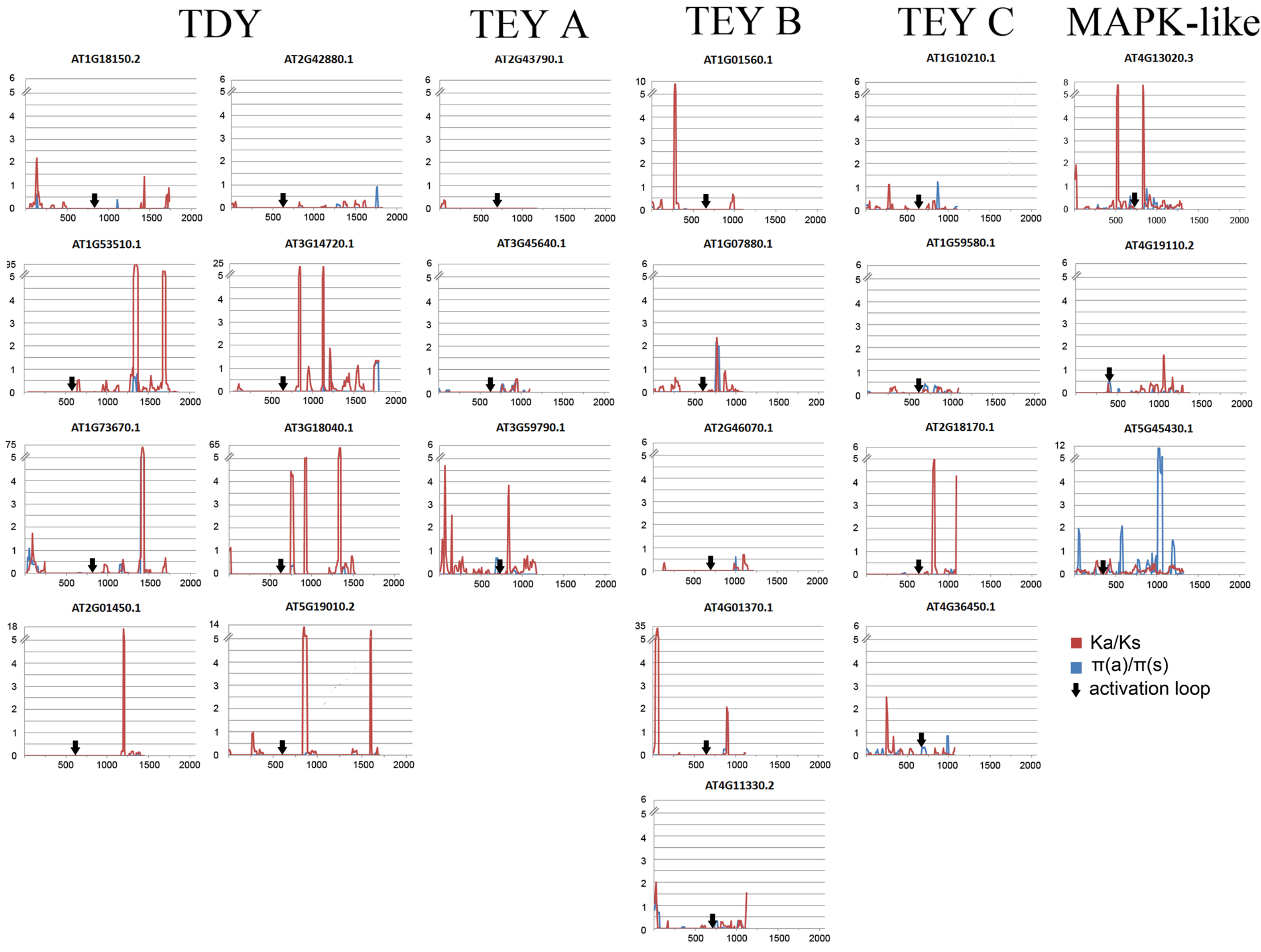

TDY
TEY A
TEY B
TEY C
MAPK-like
AT1G18150.2
AT2G42880.1
AT2G43790.1
AT1G01560.1
AT1G10210.1
AT4G13020.3
AT1G53510.1
AT3G14720.1
AT3G45640.1
AT1G07880.1
AT1G59580.1
AT4G19110.2
AT1G73670.1
AT3G18040.1
AT3G59790.1
AT2G46070.1
AT2G18170.1
AT5G45430.1
AT2G01450.1
AT5G19010.2
AT4G01370.1
AT4G36450.1
AT4G11330.2
Ka/Ks
π(a)/π(s)
activation loop

Table S1: Major types of MAPKs in plant genomes.

Group	*Organism*	*Gene-ID*	*Name*
**TEY A**	*A. lyrata*	484948	AlMPK3
		867420	
		903782	
	A. thaliana	AT2G43790.1	AtMPK6
		AT3G45640.1	AtMPK3
		AT3G59790.1	AtMPK10
	B. distachyon	Bradi1g49100.1	
		Bradi1g65810.1	
	C. papaya	evm.model.supercontig_139.47	
		evm.model.supercontig_343.2	
		evm.TU.contig_47666.1	
	G. max	Glyma02g15690.1	
		Glyma07g32750.1	
		Glyma11g15700.1	
		Glyma12g07770.1	
	O. sativa	LOC_Os03g17700.1	OsMPK3, OsMPK5
		LOC_Os06g06090.1	OsMPK6,Os MPK1
	P. trichocarpa	POPTR_0001s27860.1	PtMPK3.1
		POPTR_0007s00930.1	PtMPK6.1
		POPTR_0009s07050.1	PtMPK3.2
		POPTR_0017s04290.1	PtMPK6.2
	S. bicolor	Sb01g038750.1	
		Sb10g003810.1	similar to MPK1
	V. vinifera	GSVIVT01025105001	
		GSVIVT01038192001	
	Z. mays	GRMZM2G002100_T01	ZmMPK7
		GRMZM2G017792_T01	
		GRMZM2G020216_T01	ZmMPK5
		GRMZM2G053987_T02	
**TEY B**	*A. lyrata*	322261	
		878970	
		887292	AtlMAPK 11
		911474	AlMAPK 5
		912096	AlMAPK 4
	A. thaliana	AT1G01560.2	AMPK11
		AT1G07880.2	AtMPK13
		AT2G46070.1	AtMPK12
		AT4G01370.1	AtMPK4
		AT4G11330.1	AtMPK5
	B. distachyon	Bradi3g16560.1	
		Bradi3g32000.1	
	C. papaya	evm.model.supercontig_139.35	
		evm.model.supercontig_184.17	

	G. max	Glyma01g43100.1	
		Glyma05g37480.1	
		Glyma07g07270.1	
		Glyma08g02060.1	
		Glyma09g39190.1	
		Glyma11g15590.1	
		Glyma16g03670.1	
		Glyma18g47140.1	
		Glyma12g07850.1	
	O. sativa	LOC_Os08g06060.1	OsMPK2
		LOC_Os10g38950.1	OsMPK4, OsMPK6
	P.patens	Pp1s149_39V6.3	
		Pp1s20_266V6.1	
		Pp1s29_285V6.1	
		Pp1s59_325V6.1	
		Pp1s99_26V6.1	
	P. trichocarpa	POPTR_0001s09800.1	
		POPTR_0002s16400.1	PtMPK4.1
		POPTR_0003s13170.1	PtMMK3.1
		POPTR_0014s08450.1	PtMMK3.2
	S. moellendorffii	105143	
	S. bicolor	Sb01g030680.1	similar to MPK6
		Sb07g003810.1	similar to MPK 2
	V. vinifera	GSVIVT01019406001	
		GSVIVT01025091001	
		GSVIVT01026984001	
	Z. mays	GRMZM2G123886_T01	
		GRMZM2G127141_T01	
**TEY C**	*A. lyrata*	319769	MPK7
		353194	MPK14
		471135	MPK1
		876031	
	A. thaliana	AT1G10210.1	AtMPK1
		AT1G59580.1	AtMPK2
		AT2G18170.1	AtMPK7
		AT4G36450.1	AtMPK14
	B. distachyon	Bradi1g34030.1	
		Bradi3g03780.1	
		Bradi4g24910.1	
	C. papaya	evm.model.supercontig_6.174	
	C. reinhardtii	Cre01.g010000.t1.1	CrMAPK8
		Cre12.g508900.t1.1	CrMAPK6
		Cre12.g509000.t1.2	CrMAPK3
	G. max	Glyma04g03210.1	
		Glyma05g28980.1	
		Glyma06g03270.1	
		Glyma08g12150.1	

	O. sativa	LOC_Os02g05480.1	OsMPK14, OsMPK3
		LOC_Os06g48590.2	OsMPK7, OsMPK4
	P. patens	Pp1s138_117V6.1	
		Pp1s207_63V6.1	
	P. trichocarpa	POPTR_0002s03340.1	PtMPK1.2
		POPTR_0005s12140.1	PtMPK7.1
		POPTR_0005s25270.1	
		POPTR_0007s13370.1	
	S. moellendorffii	75282	
		443152	
	S. bicolor	Sb04g003480.1	similar to MPK3
		Sb10g028780.1	
	V. vinifera	GSVIVT01009766001	
		GSVIVT01018883001	
	Z. mays	GRMZM2G048455_T01	
		GRMZM2G062914_T01	ZmMPK14
**TDY**	*A. lyrata*	474479	AlMPK 18
		476507	AlMPK15
		478884	AlMPK19
		483386	AlMPK20
		488785	AlMPK16
		864560	
		880114	
		898157	
	A. thaliana	AT1G18150.2	AtMPK8
		AT1G53510.1	AtMPK18
		AT1G73670.1	AtMPK15
		AT2G01450.1	AtMPK17
		AT2G42880.1	AtMPK20
		AT3G14720.1	AtMPK19
		AT3G18040.1	AtMPK9
		AT5G19010.1	AtMPK16
	B. distachyon	Bradi1g34700.1	
		Bradi1g41780.1	
		Bradi2g15320.1	
		Bradi2g15620.1	
		Bradi2g16340.1	
		Bradi2g36470.1	
		Bradi2g44350.1	
		Bradi2g45010.1	
		Bradi2g45870.1	
	C. papaya	evm.model.supercontig_3.416	
		evm.model.supercontig_50.81	
		evm.model.supercontig_65.147	
		evm.TU.contig_29215.1	
	C. reinhardtii	Cre08.g385050.t1.2	CrMAPK2
		Cre20.g759500.t1.2	CrMAPK4

	G. max	Glyma02g45630.1	
		Glyma05g33980.1	
		Glyma07g11470.1	
		Glyma07g38510.1	
		Glyma08g05700.1	
		Glyma08g42240.1	
		Glyma09g30790.1	
		Glyma13g28120.1	
		Glyma13g33860.1	
		Glyma14g03190.1	
		Glyma15g10940.1	
		Glyma15g38490.1	
		Glyma17g02220.1	
		Glyma18g12720.1	
	O. sativa	LOC_Os01g43910.1	OsMPK20-1, OsMPK10
		LOC_Os01g45620.1	OsMPK21-2, OsMPK16
		LOC_Os01g47530.1	OsMPK20-4, OsMPK8
		LOC_Os02g04230.3	OsMPK17-2, OsMPK13
		LOC_Os05g05160.1	OsMPK14
		LOC_Os05g49140.1	OsMPK20-5, OsMPK7
		LOC_Os05g50120.1	OsMPK21-1, OsMPK17
		LOC_Os06g26340.1	OsMPK20-3, OsMPK11
		LOC_Os11g17080.1	OsMPK16, OsMPK15
		LOC_Os06g49430.1	OsMPK17-1, OsMPK12
	P. patens	Pp1s80_71V6.2	
		Pp1s87_157V6.1	
	P. trichocarpa	POPTR_0001s39020.1	PtTDY1.2
		POPTR_0002s06080.1	PtMPK20.2
		POPTR_0005s22350.1	
		POPTR_0008s20530.1	PtMPK16.2
		POPTR_0010s03070.1	PtMPK16.1
		POPTR_0010s12240.1	
		POPTR_0011s10390.1	PtTDY1.1
		POPTR_0012s04590.1	
		POPTR_0015s04380.1	PtMPK8.2
	S. moellendorffii	74687	
		97841	
	S. bicolor	Sb03g028740.1	similar to MPK10
		Sb03g029340.1	similar to MPK16
		Sb03g030450.1	similar to MPK8
		Sb04g002830.1	similar to MPK13
		Sb05g010000.1	
		Sb09g003280.1	similar to MPK6
		Sb09g028690.1	similar to MPK7
		Sb09g029370.1	similar to MPK17
		Sb09g029720.1	similar to MPK10
		Sb10g029400.1	similar to MPK12

	V. vinifera	GSVIVT01000784001	
		GSVIVT01008408001	
		GSVIVT01011749001	
		GSVIVT01014081001	
		GSVIVT01017873001	
	Z. mays	GRMZM2G007848_T01	
		GRMZM2G034052_T01	
		GRMZM2G062761_T01	
		GRMZM2G063144_T01	
		GRMZM2G089484_T01	ZmMPK6
		GRMZM2G122335_T01	
		GRMZM2G131334_T02	
		GRMZM2G135904_T02	
		GRMZM2G163861_T01	
		GRMZM2G306028_T02	
		GRMZM2G314048_T01	
		GRMZM2G374088_T01	ZmMPK17-2
		GRMZM2G375975_T01	
**Ancient MAPKs**	*C. reinhardtii*	Cre13.g607300.t1.1	CrMAPK5
	S. moellendorffii	82767	
**MAPK-like**	*A. lyrata*	492943	
		883637	
	A. thaliana	AT4G13020.3	MHK
		AT4G19110.2	
		AT5G45430.1	
	B. distachyon	Bradi1g01840.1	
		Bradi1g50590.1	
		Bradi3g52530.1	
	C. papaya	evm.model.supercontig_13.11	
		evm.model.supercontig_13.13	
		evm.model.supercontig_13.9	
		evm.model.supercontig_24.36	
		evm.model.supercontig_8.120	
		evm.model.supercontig_96.33	
	C. reinhardtii	Cre16.g661100.t1.1	CrMAPK7
		Cre03.g164250.t1.2	FAP262
		Cre04.g223200.t1.2	
		Cre10.g432250.t1.2	
	G. max	Glyma01g35190.1	
		Glyma09g34610.1	
		Glyma16g08080.1	
		Glyma16g17580.1	
	O. sativa	LOC_Os02g47220.1	
		LOC_Os02g47225.1	
		LOC_Os03g63020.1	
		LOC_Os06g02550.2	
		LOC_Os06g02555.1	

	P. patens	Pp1s138_154V6.1	
		Pp1s173_136V6.1	
		Pp1s28_184V6.2	
		Pp1s34_221V6.1	
		Pp1s479_13V6.1	
	P. trichocarpa	POPTR_0002s24890.1	PtMHK.1
		POPTR_0003s10090.1	
	S. moellendorffii	77240	
		443358	
	S. bicolor	Sb01g001390.1	
		Sb04g030800.1	similar to CDK F-4
		Sb10g001310.1	GAMYB-binding protein
	V. vinifera	GSVIVT01005924001	
		GSVIVT01022771001	
	Z. mays	GRMZM2G045241_T01	
		GRMZM2G108829_T01	
		GRMZM2G178822_T01	
		GRMZM5G864414_T03	
